# Dose-dependent effects of adalimumab in neonatal rats with hypoxia/reoxygenation-induced intestinal damage

**DOI:** 10.17305/bjbms.2020.4823

**Published:** 2021-02

**Authors:** Halil Kocamaz, Özmert MA Özdemir, Nilay Şen Türk, Yaşar Enli, Barbaros Şahin, Hacer Ergin

**Affiliations:** 1 Department of Pediatric Gastroenterology, Faculty of Medicine, Pamukkale University, Denizli, Turkey; 2 Division of Neonatology, Department of Pediatrics, Faculty of Medicine, Pamukkale University, Denizli, Turkey; 3 Department of Pathology, Faculty of Medicine, Pamukkale University, Denizli, Turkey; 4 Department of Biochemistry, Faculty of Medicine, Pamukkale University, Denizli, Turkey; 5 Experimental Animal Study Laboratory, Pamukkale University, Denizli, Turkey

**Keywords:** Adalimumab, hypoxia, intestine, reperfusion, tumor necrosis factor-alpha

## Abstract

Tumor necrosis factor-alpha (TNF-α) has an important role in hypoxia/reoxygenation (H/R)-induced intestinal damage. It was shown that blocking TNF-α with infliximab has beneficial effects on experimental necrotizing enterocolitis and hypoxic intestinal injury. However, there is no data about the effect of adalimumab on H/R-induced intestinal damage. Therefore, we aimed to determine potential dose-dependent benefits of adalimumab in such damage in neonatal rats. Wistar albino rat pups were assigned to one of the four groups: control group, hypoxia group, low-dose adalimumab (5 mg/kg/day) treated group (LDAT), and high-dose adalimumab (50 mg/kg/day) treated group (HDAT). On the fourth day of the experiment, all rats except for the control group were exposed to H/R followed by euthanasia. Malondialdehyde (MDA), myeloperoxidase (MPO), TNF-α, total antioxidant capacity (TAC), and total oxidant capacity (TOC) were measured in intestinal tissue. TAC and TOC values were used to calculate the oxidative stress index (OSI). Histopathological injury scores (HIS) were also evaluated in the tissue samples. MDA levels were significantly lower in the LDAT and HDAT groups (p < 0.001). TNF-α levels were significantly lower in the LDAT group (p < 0.001). OSI was significantly higher in the H/R group than in the control and LDAT groups (p < 0.001). Mean HIS values in the LDAT group were significantly lower than those in the H/R and HDAT groups (p < 0.001). This experimental study showed that low-dose adalimumab appears to have a beneficial effect on intestinal injury induced with H/R in neonatal rats.

## INTRODUCTION

Ischemia following reperfusion causes damage known as ischemia/reperfusion or reoxygenation injury in numerous internal organs, including the liver, lungs, kidneys, and intestines [1]. Ischemia leads to a pro-inflammatory condition through reactive oxygen species (ROS), inflammatory cytokines, and activation of neutrophils, and the inflammation increases tissue damage during reoxygenation injury [2]. Necrotizing enterocolitis (NEC) is acquired inflammatory bowel necrosis that leads to mortality and morbidity in the neonatal intensive care unit (NICU) [3]. Although sepsis, enteral nutrition, and deterioration of the microbiota are risk factors for this disorder, the main factor responsible for the disease is inflammation triggered by hypoxia/reoxygenation (H/R) damage in the immature bowel. The interaction between tumor necrosis factor-alpha (TNF-α) and platelet-activating factor during inflammation enhances the mucosal damage in NEC. TNF-α mRNA and protein levels are increased in the intestines of patients with NEC [4,5]. NEC occurs in 1–8% of infants admitted to the NICU. Despite the development of NICUs and advanced medical care, the prevention and management of NEC are still unsatisfactory. Medical therapies for NEC often fail, and surgical intervention may cause mortality and morbidity [3,6].

Adalimumab is an effective monoclonal antibody that blocks TNF-α and is clinically indicated in several rheumatological and inflammatory bowel diseases [7]. Some studies have shown that blocking TNF-α has beneficial effects on NEC and ischemia/reperfusion-induced intestinal damage [8]. However, no studies have investigated the effects of adalimumab on H/R-induced intestinal damage or NEC. Several experimental studies investigated the effectiveness of adalimumab at different doses. In those studies, adalimumab as an anti-inflammatory agent significantly decreased nerve tissue lipid peroxidation in rats with experimental peripheral nerve injury, and it had beneficial effects on experimentally induced acute pancreatitis in rats [9,10]. The current study aimed to determine dose-dependent effects of adalimumab on intestinal injury induced with H/R in neonatal rat pups.

## MATERIALS AND METHODS

### Study design

Twenty-eight neonatal Wistar albino rat pups aged one day (mean weight 6 g) were randomly assigned to one of four groups, each containing seven animals. The mothers were fed pellets under standard conditions. Group I, the control group, was not treated or exposed to H/R. Group II, the hypoxia group, was also untreated but was exposed to H/R. Group III, the low-dose adalimumab treated (LDAT) group, was also exposed to H/R. In this group, adalimumab was administered subcutaneously (s.c.) at a dosage of 5 mg/kg/day. Group IV, the high-dose adalimumab treated (HDAT) group, was also exposed to H/R and received adalimumab s.c. at a dosage of 50 mg/kg/day.

### H/R procedure design

All rat pups were subsequently returned to their mothers under normothermic conditions (22–23°C). Adalimumab (Humira, 40 mg/0.8 ml AbbVie Laboratories, Istanbul, Turkey) was administered s.c. at 5 mg/kg/day to Group III and at 50 mg/kg/day to Group IV over 3 days. The rat pups in Groups I and II received saline injections s.c. for the same 3-day period. In the next stage of the experiment, the 4-day-old pups in Groups II, III, and IV were exposed to H/R following the procedure described by Okur et al. [11]. Briefly, the pups were placed into an airtight Plexiglas chamber containing 100% CO_2_ for 5 min. By the end of this time, the pups were observed to be cyanotic and gasping. Following hypoxia induction, the pups were reoxygenated with 100% oxygen over 5 min. All rat pups were euthanized on a postnatal day 4 by intraperitoneal injection of pentobarbital sodium overdose 6 h after the H/R procedure [12].

### Sample collection

After the abdominal dissection of rat pups, the whole of the intestine was removed. The cecum was also observed; then, the last 2 cm section of the small intestine was identified as the distal ileal segment. Distal ileal segments were excised from all rat pups for histopathological and biochemical assessments. Intestinal tissue samples were stored at -70°C for biochemical analysis, and all biochemical assessments were performed at 20°C. Histopathological injury score (HIS), malondialdehyde (MDA), myeloperoxidase (MPO), TNF-α, total antioxidant capacity (TAC), and total oxidant capacity (TOC) were measured in the intestinal tissue samples.

### Histopathological assessment

A section of the distal ileum collected from each rat pup was fixed in 10% buffered formalin, placed in a paraffin block, sectioned at 5 mm, and stained with hematoxylin and eosin for histological evaluation. HIS was graded by the same blinded pathologist. Grade I was defined as normal histological architecture, Grade II as (minimal) hydropic degeneration and/or the presence of surface epithelial disturbance originating from the lamina propria, Grade III as (mild) restricted epithelial cell necrosis in the villus tips, Grade IV (moderate) as observation of complete necrosis in villi, and Grade V (severe) was defined as transmural necrosis [13].

### Measurement of TNF-α

The terminal ileal tissues were placed in Eppendorf tubes and stored at -80°C until assayed. TNF-α levels were measured using an enzyme-linked immunosorbent assay (ELISA) kit (Invitrogen/Thermo Fisher, Camarillo, CA, USA). TNF-α levels were expressed as pg/ml.

### Measurement of TAC

Intestinal TAC levels were detected using a novel automated measurement method developed by Erel (Rel Assay Diagnostics kit; Mega Tıp, Gaziantep, Turkey). This technique is based on measuring the antioxidant effect exhibited by the sample against potent free radical reactions triggered by hydroxyl radicals. The results obtained were expressed as mmol Trolox Eq/L [14].

### Measurement of TOC

Intestinal TOC values were measured using the novel automated technique described by Erel (Rel Assay Diagnostics kit; Mega Tıp). Briefly, color intensity measured using spectrophotometric analysis is calculated according to the total oxidant molecules contained in that sample. Hydrogen peroxide (H_2_O_2_) was used for calibration, and the results were expressed as micromolar hydrogen peroxide equivalents per liter (mmol H_2_O_2_ Eq/L) [15].

### Calculation of oxidative stress index (OSI)

The OSI was defined as the ratio of TOC to TAC (OSI [arbitrary units] = TOC [μmol H_2_O_2_ Eq/L]/TAC [μmol Trolox Eq/L]) [16].

### Measurement of MDA

Intestinal MDA levels were measured with an ELISA kit (Shanghai YL Biotech Co. Ltd., Shanghai, China). The absorbance was recorded at 532 nm and subsequently compared with the comparable values of MDA standards. The MDA results were expressed as nmol/ml [17].

### Measurement of MPO

MPO levels in the intestinal tissues were measured using an ELISA kit (Cusabio, Wuhan, China). The absorbance was recorded at 450 nm and subsequently compared with those of MPO standards. The results obtained were expressed as ng/ml [18].

### Ethical statement

The approval for this experimental study was granted by the Pamukkale University Animal Research Committee (approval number: PAUHADYEK-2017/05).

### Statistical analysis

Continuous data were expressed as mean ± standard deviation or median (range), and categorical data were expressed as numbers. Normality assumptions were examined using the Shapiro–Wilk test. One-way analysis of variance (ANOVA) and the Bonferroni post-hoc test were used to compare biochemical parameters in tissue specimens. The Mann–Whitney U test was used to compare HIS values between the groups. Values of *p* < 0.05 were considered statistically significant.

## RESULTS

### Histopathological changes in the rat intestine

**FIGURE 1:**
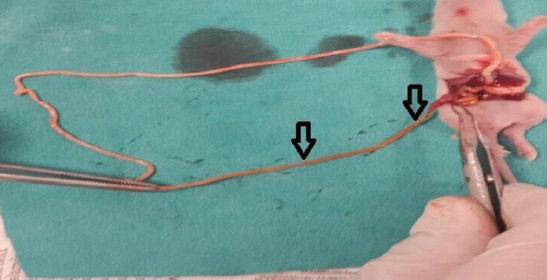
Abdominal dissection and determination of the distal ileal segment.

**FIGURE 2:**
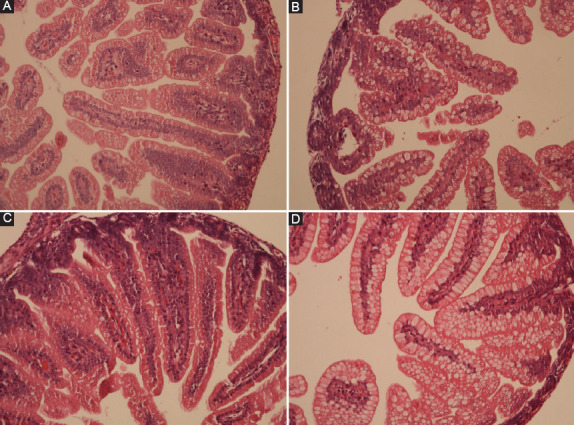
Histopathological images of the rats’ intestines (H and E, ×200). (A) Control group (Group I), rat number 7, normal histology, HIS: 1; (B) hypoxia group (Group II), rat number 2, epithelial hydropic degeneration, HIS: 2; (C) low-dose adalimumab group (Group III), rat number 6, normal histology, HIS: 1; (D) high-dose adalimumab group (Group IV), rat number 2, epithelial hydropic degeneration, HIS: 2. HIS: Histopathological injury score.

In this experimental study, only two rat pups from Group II died after the H/R procedure. Necropsy revealed hemorrhaging, discoloration, and perforations in the intestines. Macroscopic abdominal examination of the other rat pups in the Group II (H/R), III (LDAT), and IV (HDAT) revealed similar findings, but without intestinal perforations. Abdominal dissection and examination of the whole intestine of rat pups are shown in Figure 1. The histopathological appearances of the groups are shown in Figure 2. The main histological finding in the H/R and HDAT groups was epithelial hydropic degeneration, grade II HIS (Figure 2B and D). The mean HIS in the LDAT group was significantly lower than in the H/R and HDAT groups (HIS 1.14, 1.84, and 1.86 in the LDAT, H/R, and HDAT group respectively; *p* < 0.001). The mean HIS values of the study groups are given in Table 1.

**TABLE 1:**
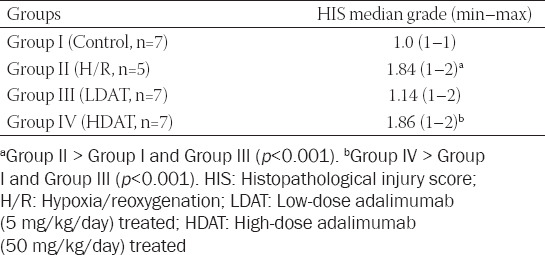
Histopathological injury scores of the study groups

### Determination of MDA, MPO, and TNF-α levels in the intestinal tissue

Intestinal MDA levels were significantly lower in the LDAT and HDAT groups compared to the H/R group (*p* < 0.001). MPO levels were significantly higher in the H/R group than in the control group (*p* < 0.001), but no differences were observed among the other groups (*p* > 0.05). TNF-α levels in the H/R group were significantly higher than those in the control and LDAT groups (*p* < 0.001), whereas TNF-α levels in the HDAT group were significantly higher than those in the control and LDAT groups (*p* < 0.001). Intestinal tissue MDA, MPO, and TNF-α levels of the groups are shown in Table 2.

**TABLE 2:**
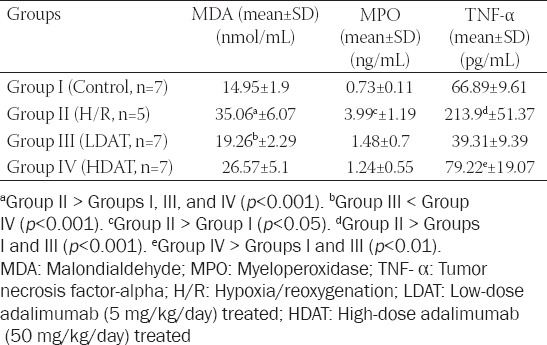
Biochemical evaluation of MDA, MPO, and TNF-α levels in the study groups

### Determination of TAC and TOC levels and the calculated OSI values

The highest TAC level was determined in the control group. Significantly lower TAC levels were observed in the H/R group compared to the control and LDAT groups (*p* < 0.001), while TOC levels were significantly higher in the H/R group compared to the control group (*p* < 0.05). No significant differences were determined among the other groups (*p* > 0.05). OSI in the H/R group was significantly higher than in the control and LDAT groups (*p* < 0.001), and OSI in the HDAT group was significantly higher than in the control group (*p* < 0.001). The mean TAC, TOC, and OSI values of the groups are shown in Table 3.

**TABLE 3:**
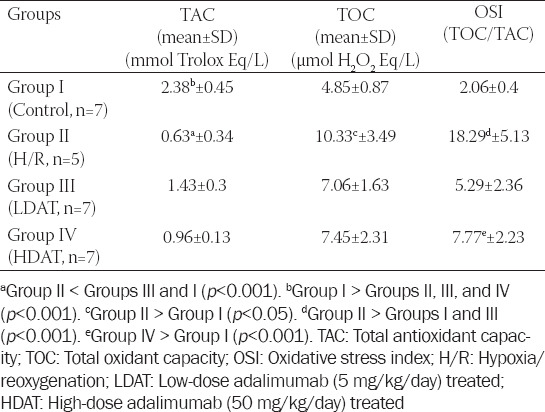
Biochemical evaluation of TAC, TOC levels, and OSI in the study groups

## DISCUSSION

The purpose of this experimental study was to investigate the dose-dependent therapeutic effects of adalimumab on intestinal injury induced with H/R in a neonatal rat pup model. To the best of our knowledge, this is the first study that evaluated the effect of adalimumab on H/R-induced intestinal injury. Our findings showed that treatment with low-dose (5 mg/kg/day) adalimumab attenuated the intestinal injury in neonatal rats. However, similar beneficial effects on the intestinal injury were not observed at a higher dose of adalimumab (50 mg/kg/day).

Greater than 90% of cases with NEC occur in premature infants and the incidence of this disease increases dramatically in the smallest and most premature infants [19]. Although the etiology of the disease is still unclear, hypoxia and ischemia are known to play an important role in the pathogenesis [20]. The intestines of premature newborns are particularly susceptible to ischemia due to incomplete development of the microvasculature and inadequate supply of O_2_ to the intestinal tissue [21]. A 1-day-old rat corresponds to a human fetus at about 22- to 24-week gestation and a 3-day-old rat to a human fetus at about 28 to 32 weeks [22,23]. Therefore, we designed this study to investigate the dose-dependent therapeutic effects of adalimumab on the biochemical and histopathological alterations in 1- to 4-day-old rat pups exposed to H/R. Okur et al. reported that histopathological lesions in neonatal rats exposed to H/R were similar to those found in early NEC [11]. The same method of H/R was used in other studies [2,12]. Previous studies in rats induced with H/R showed that the histopathological lesions ranged from normal histology to transmural necrosis and that the prominent microscopic lesions were located in the distal intestinal segment [2,11-13]. Therefore, the method described by Ozkan et al. [13] was used to assess the histopathological findings in the current study. Inflammatory mediators and oxygen-derived free radicals are well-known to play roles in the progression of H/R-induced intestinal injury [12]. TNF-α has been shown to stimulate its own production as well as the production of other chemokines and cytokines [5]. TNF-α blockade, therefore, plays a critical role during the inflammatory process and, theoretically, during NEC. Another TNF-α blocking agent, infliximab, was reported to attenuate intestinal injury by reducing lipid peroxidation and oxidative stress in an experimental NEC model [8]. Low-dose adalimumab (5 mg/kg/day) was observed to exhibit ameliorative effects on the intestinal injury induced through H/R in the present study, whereas the high dose of adalimumab (50 mg/kg/day) did not exhibit similar properties. Polat et al. also showed that low-dose adalimumab (5 mg/kg) has beneficial effects on ischemia/reperfusion-induced peripheral nerve injury, whereas a high dose of adalimumab (50 mg/kg) had no similar effects [10]. In the previous studies with rats, adalimumab was used at different low doses such as 5 mg/kg and 10 mg/kg, and at a higher dose such as 50 mg/kg [9,10,24]. In humans, adalimumab has been used as a 40 mg single bolus dose through subcutaneous injection. It is known that the absorption and distribution of adalimumab following a single dose are relatively slow [25]. To investigate the most effective therapeutic adalimumab dose for H/R-induced intestinal damage we preferred to use the drug at the dose of 5 mg/kg and 50 mg/kg for 3 consecutive days. No previous studies have described the dose-dependent effects of adalimumab on H/R-induced intestinal injury. In our study, high-dose adalimumab did not exhibit the same beneficial effect on the intestinal injury induced through H/R as the low dose. Biological agents, such as adalimumab, may also activate T lymphocytes and stimulate apoptosis [26]. The effect observed with high-dose adalimumab may be related to the activation of T lymphocytes and stimulation of apoptosis.

ROS generated by TNF-α in H/R-induced injury appear to play a critical role in tissue damage in the immature intestine [5]. Blocking TNF-α attenuates tissue damage by reducing the production of ROS [27]. We determined that hypoxia led to high levels of TNF-α and increased OSI in the intestinal tissue samples. Adalimumab, especially at the low dose, reduced TAC levels, and the OSI values suggested attenuated oxidative stress and a contribution to the healing of damaged tissue in our study. Interestingly, no difference was observed in TOC levels between the adalimumab groups. However, the TOC levels in those groups were lower than in the H/R group. Activated neutrophils migrate to the inflamed area in the intestines, leading to tissue damage through the production of ROS, respiratory burst and toxic enzymes, such as MPO. Measuring MPO activity in intestinal tissue, therefore, reflects neutrophil migration and infiltration [28]. Blocking TNF-α prevents neutrophil migration and infiltration and attenuates tissue injury by reducing toxic enzymes, such as MPO [4]. MDA levels also increase in ischemic tissues, acting as a marker of lipid peroxidation and oxidative stress [29]. In our study, the low and high doses of adalimumab both significantly reduced intestinal tissue MDA levels, which may be related to the blockage of TNF-α; this blockage, in turn, alleviated the inflammatory process and reduced lipid peroxidation. Although no significant differences were observed in MPO levels between the low-dose adalimumab (Group III) and high-dose adalimumab (Group IV) treated groups, MPO levels in the H/R group were higher than those in the adalimumab groups. This finding suggests that the blockage of TNF-α reduced neutrophil migration and infiltration. As a limitation of this study, apoptosis was not evaluated.

## CONCLUSION

This experimental study showed that adalimumab administration, particularly at the low dose (5 mg/kg/day), significantly decreased HIS, MDA, TNF-α levels, and OSI, while it increased TAC. However, the high-dose adalimumab was not found to be significantly effective on the levels of MPO, TNF-α, TAC, TOC, and OSI. The high-dose adalimumab significantly increased HIS, but it only significantly decreased MDA levels. We, therefore, conclude that the low-dose ­adalimumab has beneficial effects on the intestinal damage in H/R-induced injury in neonatal rats. Prophylactic low-dose adalimumab therapy prevents the intestinal tissue damage. However, further research is now needed to corroborate our results and to provide a better understanding of the mechanisms responsible for the protective effects of this drug on H/R-induced intestinal injury.
